# Supplementary material for: Deletion of wfs1 Impairs Oligodendrocyte Precursor Cells Dorsal Distribution and Myelination Through the wfs1-hmgcs1 Axis in Zebrafish
Source: Biology (Basel). 2026 Aug 16;15(16):1402. doi: 10.3390/biology15161402 (PMC13510151; doi:10.3390/biology15161402)
Supplement: Supplementary file 1 [file biology-15-01402-s001.zip › File S1-Transcriptome sequencing data and differentially expressed genes (DEGs) in wfs1-- zebrafish at 5 dpf/2.KEGG/wfs1KOvsWT/UP/src/dre00770.html]

dre00770


- dre:406789

- Up regulated genes

406789(1.01145231875249)

- dre:406789

- Up regulated genes

406789(1.01145231875249)

- dre:406789

- Up regulated genes

406789(1.01145231875249)

- dre:368239

- Up regulated genes

368239(1.14034759711527)

Close
